# A digital twin approach for transforming prescription drug labeling

**DOI:** 10.3389/fphar.2026.1717309

**Published:** 2026-04-10

**Authors:** Holly Kimko, Edwin Kimko, Sai Phanindra Venkatapurapu

**Affiliations:** 1 Systems Medicine, Clinical Pharmacology and Safety Sciences, AstraZeneca, Gaithersburg, MD, United States; 2 WellSpan York Hospital, Family Medicine, York, PA, United States

**Keywords:** digital twin, drug labeling, MIDD, precision medicine, quantitative systems pharmacology

## Abstract

Drug development entails extensive data collection by sponsors throughout research and development, followed by regulatory review, culminating in essential yet static prescription drug labeling. As study results are typically presented as isolated summaries, the label can underrepresent the drug’s integrated effects across biological systems. We propose that sponsors submit a Digital Twin-based companion app alongside traditional documentation to enable a more comprehensive understanding of the investigational drug for review and, after approval, to support personalized prescribing in clinical practice. The development and implementation of the companion app would proceed by aligning with emerging regulatory guidance and by leveraging robust, scalable technical architecture.

## Introduction

The journey of a pharmaceutical compound from laboratory to patient is a complex and meticulously documented process, culminating in regulatory approval and the dissemination of crucial information via human prescription drug labeling. This document, as specified by FDA’s Physician Labeling Rule format (U.S Food and Drug Administration, 2013), provides essential details on indications, dosage, contraindications, potential adverse events, and other critical information for the safe and effective use of a drug. The content of drug labeling is substantiated by extensive data compiled from numerous nonclinical and clinical studies in regulatory submission packages such as the New Drug Application (NDA) and the Biologics License Application (BLA). However, while sponsors often integrate evidence across studies to support labeling, much of the submitted summaries and analyses still appear as isolated, non-integrated entities, limiting a holistic view of the drug’s system-level effects. This evidence-by-evidence approach, which is necessary for validating individual findings, can inadvertently hinder a comprehensive understanding of a drug’s overall impact on intricate biological systems involved in a disease.

Each *in vitro*, *in vivo*, and clinical study - with its specific design, subjects (e.g., stem cell-derived systems, animals, or patients), and endpoints - provides distinct and complementary insights into the multifaceted effects of a drug candidate. The absence of a mechanism to integrate these disparate datasets into a cohesive, system-level characterization can obscure critical insights into the drug’s interaction with underlying disease mechanisms and how these interactions might vary across individuals with different biological profiles. This limitation in current regulatory submissions highlights the need for innovative approaches that can synthesize the extensive nonclinical and clinical data to provide a more holistic and, ultimately, more personalized understanding of drug effects in individual patients.

Therefore, we propose complementing static prescription drug labeling with a Digital Twin (DT)-based companion application that delivers dynamic insights. We distinguish two instantiations of the DT that share a common simulation model. During drug development and regulatory review, the DT operates as an integration platform that consolidates multi-scale evidence, enabling simulation and hypothesis testing to inform study design and labeling. After approval, a rigorously validated clinical instantiation of the same application functions as a recommendation and scenario testing software within a Clinical Decision Support System (CDSS) (Venkatapurapu et al., 2024b), producing patient-specific dosing and treatment recommendations, for a physician to review, under defined safety guardrails. In this proposed framework, the DT drug labeling companion application is a software that uses a virtual representation of individual patients to guide personalized recommendations for safe and effective use of a drug. This software application, developed based on the totality of data collected during drug development and submitted with the regulatory package, would be positioned to advance precision medicine by translating integrated mechanistic understanding into actionable, patient-specific guidance for a physician to consider at the point of care.

## Development of a digital twin application

**FIGURE 1:**
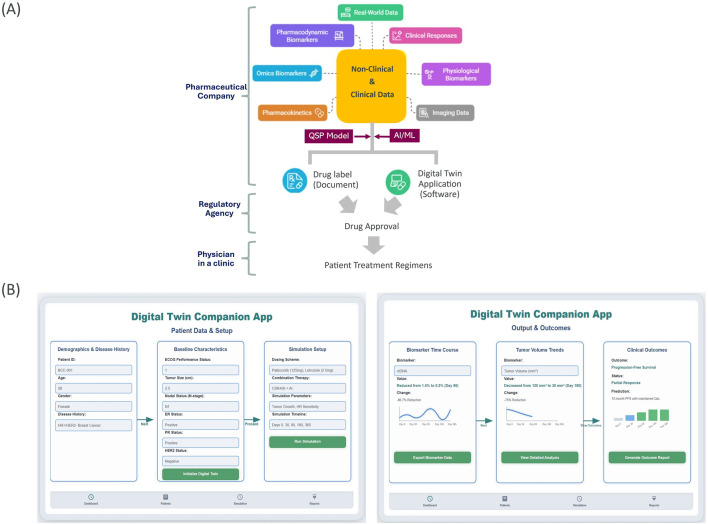
Digital Twin (DT) companion app for drug labeling. **(A)** Outlines the development workflow for a QSP/AI/ML model-based DT companion app. Different data collected during drug development are integrated into the model. These data also form the basis for the static paper drug label whereas the model powers the DT-based dynamic drug label. Both the drug labels are submitted for regulatory review and are used by physicians after regulatory approval of the drug. Drawing assisted by napkin.ai. **(B)** Shows a sample schematic of the app showing the setup screen and output dashboard.

One of the prerequisites for a DT application is a simulation model that predicts the dynamics of the real-world counterpart (Venkatapurapu et al., 2024a). The predictive model could either be created using a mechanistic approach or a machine learning approach or both. Quantitative Systems Pharmacology (QSP) offers a powerful framework for the creation and application of sophisticated computational models (Gadkar et al., 2016) using a mechanistic approach. QSP modeling excels at integrating physiological knowledge with diverse data modalities (e.g., omics profiles, spatial digital imaging data, physiological biomarkers, pharmacokinetic and pharmacodynamic data, and real-world data), often generated from disparate nonclinical/clinical investigations and healthcare settings. Acknowledging the inherent complexity of human biology, a perfectly identical DT remains currently unattainable. However, artificial intelligence and machine learning technologies offer promising avenues to complement missing biological information (Venkatapurapu et al., 2022; Folguera-Blasco et al., 2024) as our biological understanding continues to evolve. Figure 1A illustrates the workflow to develop a hybrid QSP/AI/ML model-based DT application.

By constructing a comprehensive disease platform model grounded in the understanding of physiology, pathology, and pharmacology, QSP models enable the evaluation of a drug’s systemic effects. This allows for predicting efficacy and safety endpoints with a more integrated perspective that reflects patient-specific disease status at baseline and during treatment. The traditional approach of viewing individual nonclinical and clinical studies in isolation, akin to “seeing the tree without seeing the forest,” inherently limits our ability to grasp the holistic picture of a compound’s pharmacological effects. In contrast, this platform approach facilitates a comprehensive understanding across biological scales, from molecular to organism levels. This QSP approach is explicitly recognized as a model-informed drug development (MIDD) methodology in the recent FDA M15 MIDD Guidance (U.S Food and Drug Administration, 2024a) in addition to the population pharmacokinetic/dynamic approach that quantifies inter- and intra-individual variability.

Collecting extensive clinical biomarker data is often necessary to enable a nuanced comprehension of treatment effects in patient subgroups. Such data may also illuminate potential responders previously concealed in conventional analyses. Moreover, QSP models based on those biomarker time courses contribute significantly to achieving a balance between safety and efficacy by identifying optimal dosage regimens and patient populations that manifest a favorable risk-benefit profile. Ultimately, the insights gleaned from QSP model-based DT applications establish a critical foundation for supporting precision medicine claims, driving the development of tailored therapeutic interventions that better serve diverse patient needs. Figure 1B illustrates a schematic of a dynamic drug labeling DT companion application that a clinician could use to better understand the effects of the drug in individual patients. The application could provide insights into drug-drug interactions, safety, and efficacy based on the individual patient’s physiology, disease history, comorbidities and concomitant medications. Recognizing the critical impact of DT predictions on patient safety and treatment efficacy, pharmaceutical sponsors should prepare robust DT applications for thorough regulatory assessment of their validity and reliability during drug approval processes.

## Digital twins for a holistic view of nonclinical data

Integrating QSP model-based DTs significantly enhances the regulatory review of nonclinical data in Investigational New Drug Applications (INDs), not limited to BLAs or NDAs for drug labeling. This approach provides a comprehensive understanding of an investigational drug’s effects across biological scales - from molecular interactions to animal responses – supporting more reliable translation to human biology within the DT application. By dynamically depicting drug effects and simulating clinically relevant dose-response relationships, QSP models improve predictions of how dosage adjustments may influence efficacy and safety across patient subpopulations, thereby aiding clinical study design decisions. The DT, developed with nonclinical responses from human-relevant *in vitro* and *in vivo* models and progressively refined with clinical response data as it becomes available, can be adapted to support related drug modalities or adjacent disease indications by extending the shared mechanistic framework and calibrating to modality- or indication-specific datasets.

## Integrating various clinical biomarker data to personalize digital twins

Clinical outcome data is most relevant for substantiating a drug’s efficacy and safety in labeling. Beyond traditional statistical and pharmacometric analyses, mechanistic QSP modeling refines clinical study interpretations, providing a more complete understanding of drug effects in human subjects. DT applications offer a robust platform to fuse heterogeneous clinical datasets, integrating primary clinical outcome data alongside critical pharmacodynamic markers and surrogate endpoints. In addition, while quantifying drug exposure at the effect site often presents challenges, QSP models can predict local exposure and its downstream effects, improving understanding of clinical responses and optimizing treatment choices for specific subpopulations, including apparent non-responders (Venkatapurapu et al., 2022).

Furthermore, integrated QSP models significantly facilitate the early and efficient identification of potential safety signals. These models mathematically connect cellular-level mechanisms with whole-organism responses, leveraging combined insights from *in vitro*, *in vivo*, and clinical studies to simulate system-wide drug effects (Goldring et al., 2025). This holistic perspective is crucial for uncovering off-target interactions and potential toxicities often missed by reductionist studies. By predicting these risks in the drug development pipeline, QSP models contribute to more informative and accurate drug safety profiles, enhancing drug labeling content, and ultimately bolstering patient safety in the clinic by facilitating more informed clinical decision-making.

## Application of digital twin in clinical decision support systems

**FIGURE 2:**
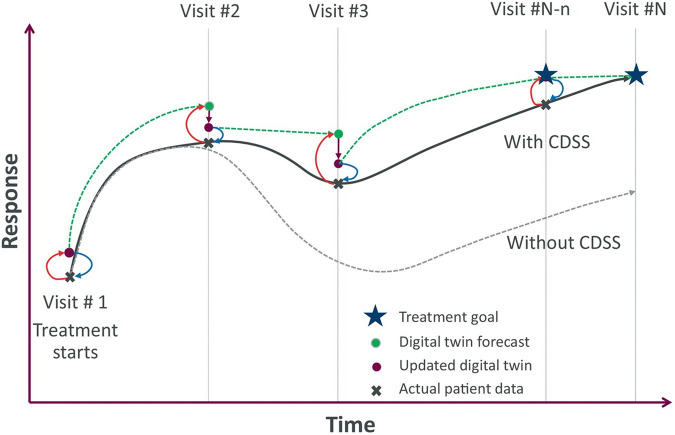
Illustrative trajectories of potential DT-based clinical decision support system for achieving treatment goal. This conceptual diagram shows two scenarios. In the first scenario, a patient reaches their treatment goal (solid black line) when their physician uses a DT-based clinical decision support system (CDSS) to optimize the treatment plan at each visit. In the second scenario, a patient starts well but veers off track and the physician lacks a CDSS to guide the patient back on track (dotted black line). The figure legend describes what each marker represents. Green dotted lines represent the time course trajectory of the DT forecast between consecutive visits. Red arrows represent the feedback from the patient’s condition during the current visit to update the DT. Blue arrows represent insights from the DT, either newly created or updated during the current visit, to optimize the treatment plan. Reprinted from (Venkatapurapu et al., 2024b).

Incorporation of DTs within CDSS holds immense potential for transforming healthcare (Figure 2) (Venkatapurapu et al., 2024b). With regulatory approval of such applications, whenever applicable, CDSS can transcend population-based guidelines and offer patient-specific dosage regimen recommendations by leveraging the predictive capabilities of DTs. For instance, a DT application could simulate the effects of various drug dosages and treatment combinations on a particular patient, considering their unique physiological characteristics and disease state at the onset of treatment and throughout its course. This capability allows clinicians to anticipate potential adverse events, optimize treatment plans to maximize efficacy and ultimately make more informed decisions at the point of care. Preliminary research suggests that this approach has the potential to significantly enhance the precision and effectiveness of medical interventions (Venkatapurapu et al., 2022). However, it is critical to note that the DT application is designed to augment (Venkatapurapu et al., 2024b), not replace, clinical decision-making. The software provides predictions based on the patient’s current state and potential treatment scenarios, but the responsibility for treatment decisions remains with the clinician. The outputs are explanatory, offering insights into the potential outcomes of different treatment choices, and are not intended as directives.

## Regulatory strategy for a DT-based drug label companion application

DT-based drug labeling will require a practical, coordinated regulatory approach. Two interconnected aspects define the regulatory strategy – the classification of a DT companion application and the pathway for review and approval. The regulatory classification of DT applications depends on the intended use. During the pre-market regulatory review stage, the intended use is to integrate evidence from multiple sources including the studies conducted during drug development to provide a comprehensive assessment of the safety and efficacy of a novel drug. When scoped and implemented accordingly, the DT companion application can qualify as a *non-device clinical decision support* (CDS) tool under FDA’s updated CDS guidance (Food and Drug Administration, 2026), rather than as *software as a medical device* (SaMD) (U.S Food and Drug Administration, 2018).

Under the updated CDS guidance, a DT companion application qualifies as non-device CDS, and is exempt from regulation, when all four statutory criteria (Food and Drug Administration, 2026) are met:

1. *Criterion 1 (data acquisition): The software is not intended to acquire, process, or analyze a medical image or a signal from an in vitro diagnostic device or a pattern or signal from a signal acquisition system.* This criterion is satisfied for a label companion that runs population or virtual cohort simulations using prespecified study inputs and published or unpublished non-clinical and clinical data and does not ingest raw physiological signals.
2. *Criterion 2 (information handled): The software is intended for the purpose of displaying, analyzing, or printing medical information about a patient or other medical information.* A labeling companion typically involves summarizing approved label evidence, trial data, modeled exposures, and contextualized risk–benefit. A critical condition is that the tool should not be designed to consume a continuous stream of physiological data (e.g., real time wearable feeds). Continuous ingestion and processing of physiological signals may implicate Criterion 1 and push the product toward SaMD, especially if those signals are transformed for patient specific inference.
3. *Criterion 3 (role in clinical care): The software is intended for the purpose of supporting or providing recommendations to a healthcare provider (HCP) about prevention, diagnosis, or treatment of a disease or condition.* The software should not be intended to replace HCP’s decision making. The software outputs should be explanatory, scenario based, and non-directive, and they should avoid real time titration or emergent care recommendations because automation bias increases when users lack sufficient time to consider other information.
4. *Criterion 4 (independent review): The software is intended for the purpose of enabling an HCP to independently review the basis for the recommendations that such software presents so that it is not the intent that the HCP rely primarily on any of such recommendations to make a clinical diagnosis or treatment decision regarding an individual patient.* For a QSP model-based DT application, subsection (c) of Criterion 4 is especially relevant, which recommends that the software and its labeling should include:
  - A plain language summary of the modeling approach (e.g., physiologically based pharmacokinetic or QSP modeling, or AI/ML methods), including the logic or methods used;
  - A description of the datasets relied upon, with transparency about representativeness for relevant subgroups and adherence to best practices (e.g., independent development/validation);
  - A summary of clinical studies and validation evidence with performance characteristics and known limitations by patient population, enabling HCPs to judge applicability.

If the companion satisfies all four criteria, it would be regulated as non-device CDS and would not be subject to FDA device oversight as SaMD. This classification will generally hold even in the post approval stage provided the tool is used by HCPs for education and shared decision making with patients. Fundamentally, the underlying philosophy of the CDS Guidelines relevant to DT software is that HCPs should have adequate time to review information before making decisions; accordingly, to be regulated as non-device CDS, the software should present recommendations for HCP review rather than issuing directive dosing or treatment instructions, and it should not support time-critical decision making.

However, if patient-level imaging data or continuous data streams such as wearable data are used, the DT application would be regulated as a SaMD and the specific class of SaMD would have to be determined based on the potential risk for patients, as per FDA’s SaMD guidance. In that case, the software would need to meet applicable device requirements, including premarket submission and the appropriate quality and post-market controls.

Because submitting a DT companion alongside an NDA/BLA is novel, there is currently no codified pathway for coordinated review between Center for Drug/Biologics Evaluation and Research (CDER/CBER) and Center for Devices and Radiological Health (CDRH). Consequently, a pragmatic operational model may be parallel submissions if the application is classified as SaMD (i.e., an NDA/BLA to CDER/CBER and a SaMD submission to CDRH), supported by cross-center consultation to ensure alignment between the drug labeling and the device’s intended use, claims, and safety guardrails. Alternatively, sponsors could leverage principles used for combination product coordination (U.S Food and Drug Administration, 2024b) to structure an integrated review plan, even if the companion software is not a formally designated combination product. Early and frequent pre-submission interactions would be beneficial to align on the regulatory classification for the product (non-device CDS vs SaMD), coordinated review plan (e.g., Type B/C meetings for the NDA/BLA and Q-submissions for the SaMD), synchronized timelines, and shared evidentiary expectations across centers for clinical validity, human factors, and labeling consistency. If the product is classified as SaMD, sponsors should be prepared with risk appropriate validation, including clinical performance studies where needed.

Post-approval lifecycle management also depends on the software classification. For non-device CDS, sponsors should implement robust governance and transparency practices that are consistent with the CDS framework: disciplined version control, change logs that document updates to data sources and model assumptions, clear revision dates in the user interface, and revalidation triggers tied to new evidence, label changes, or detected performance drift to satisfy Criteria 4 of FDA’s updated CDS guidelines (Food and Drug Administration, 2026). For SaMD, lifecycle management follows established device requirements, including Quality System Regulation (21 CFR Part 820), design controls, risk management (ISO 14971), usability engineering (IEC 62366), and software lifecycle processes (IEC 62304). Regardless of the regulatory classification, post-market activities should include real-world performance monitoring, model update governance with rigorous version control and traceability, periodic safety and performance reporting, and ongoing bias monitoring across diverse populations. In essence, for predictive modeling components within the DT companion app (e.g., QSP or AI/ML models), sponsors should maintain traceability from model specifications and parameters to underlying data sources. Additionally, sponsors should document verification and validation, and provide accessible summaries of the model’s scope, performance, and limitations, all in a manner that HCPs can review and understand.

## Discussion

We envision a rapidly approaching future where traditional static drug labeling is complemented by interactive DT applications that enable comprehensive regulatory review and allow simulations of an individual patient’s potential response to a treatment based on their unique biological characteristics. QSP models grounded in physiology allow physicians to explore different treatment options and perform virtual experiments using DTs of patients. By inputting patient-specific data, including disease state, comorbidities, prior and concomitant treatments, clinicians could leverage the DT to personalize treatment plans, predict individual responses, and engage *in silico* monitoring of therapeutic effects (Polasek et al., 2019).

The validation of a DT application would require a multi-faceted approach. Technical validation would ensure the model’s mathematical and computational integrity, while clinical validation would assess its predictive accuracy using data from clinical trials and real-world evidence. The validation would be specific to the intended population and indication. The QSP model that forms the core of the DT application could be discussed separately with the regulators, and their feedback on the model validation plan may be solicited. Post-approval, the model’s performance would be continuously monitored, and any updates would be validated against predefined criteria and submitted to regulatory agencies. Version control and clear documentation of model changes would be maintained to ensure traceability.

For seamless integration into clinical workflows, the DT application could be designed to interface with electronic health record (EHR) systems using standard data exchange protocols (e.g., Fast Healthcare Interoperability Resources). The application would require inputs such as patient demographics, diagnosis, disease history, laboratory values, current medications, and disease-specific biomarkers. To ensure usability, the DT application would provide intuitive visualizations and plain-language explanations of the simulations, making the outputs accessible to clinicians without specialized training in QSP. Moreover, the application would be available on-demand to avoid contributing to alert fatigue for clinicians.

DT-based drug labeling represents a significant stride towards precision medicine, requiring pharmaceutical sponsors to transform extensive data into clinical tools that provide actionable, mechanism-based insights to clinicians for improved patient outcomes. However, to realize the full potential of DT-based drug labeling, there needs to be a concerted effort to overcome the inherent challenges in model development, data integration, and regulatory frameworks (Beattie et al., 2024; Niarakis et al., 2024). The regulatory strategy hinges on a disciplined definition of intended use, clear data boundaries, and transparent model/algorithm documentation. Except where the companion application ingests and processes continuous physiological signals or delivers directive, patient-specific recommendations, typically DT-based drug label companion apps can be structured to qualify as non-device CDS that are exempt from regulation, thus reducing device regulatory burden while preserving scientific rigor and clinician oversight. In cases where the DT companion app could be classified as a SaMD, early and frequent pre-submission interactions with the regulators to align on the pathway and evidentiary requirements for approval would be critical to avoid delays in product launch.

The use of DT applications also raises important ethical, operational, and liability considerations. While the clinician retains ultimate responsibility for treatment decisions, the pharmaceutical sponsor must ensure the application’s reliability and safety. To mitigate risks of sampling bias, the model must be validated across diverse patient populations, and their characteristics should be properly documented. Furthermore, when the DT application is used to guide treatment, patients should be informed about its use as part of the consent process. Ongoing monitoring and transparency of the application’s limitations are essential to maintain trust.

Although developing these model-based tools requires significant time investment and specialized resources (Pichardo-Almarza and Kimko, 2025), their long-term benefits, such as more comprehensive regulatory review, improved understanding of a patient’s disease trajectory, and effective clinical decision-making, make a compelling argument for their development. Additionally, growing access to advanced computing and novel datasets also strongly advocates for continued innovation and collaborative initiatives to routinely develop and use DT-based drug labeling.
